# Risk factors for surgical site infection following spinal surgery in Saudi Arabia: A retrospective case–control study

**DOI:** 10.1097/MD.0000000000025567

**Published:** 2021-04-30

**Authors:** Saleh Saad AlGamdi, Maha Alawi, Rakan Bokhari, Khalid Bajunaid, Abdelmoniem Mukhtar, Saleh S. Baeesa

**Affiliations:** a Department of Ophthalmology, faculty of Medicine, imam abdulrahman bin faisal university, dammam, Saudi Arabia; b Department of Medical Microbiology and Parasitology, Infection Control and Environmental Health Unit, Faculty of Medicine, King Abdulaziz University; c Neurosurgery Section, Department of Surgery; d Department of Surgery, Faculty of Medicine, University of Jeddah; e Department of Family and Community Medicine, Rabigh Faculty of Medicine, King Abdulaziz University, Jeddah, Saudi Arabia.

**Keywords:** risk factors, Saudi Arabia, spine surgery, surgical site infections, wound infection

## Abstract

Surgical site infections (SSIs) are common complications after spinal surgery that result in increased morbidity, mortality, and healthcare costs. It was estimated that SSIs after spinal surgery resulted in a 4-fold increase in health care costs. The reported SSI rate following spinal surgery remains highly variable between approximately 0.5% and 18%. In this study, we aimed to estimate the SSI rate and identify possible risk factors for SSI after spinal surgery in our Saudi patient population.

We conducted a single-center, retrospective case–control study in Saudi Arabia that included patients who developed SSIs, while the controls were all consecutive patients who underwent spinal surgery between January 2014 and December 2016. We extracted data on patient characteristics, anthropometric measurements, preoperative laboratory investigations, preoperative infection prevention measures, intraoperative measures, comorbidities, and postoperative care.

We included 201 consecutive patients in our study; their median age was 56.9 years, and 51.2% were men. Only 4% (n = 8) of these patients developed SSIs postoperatively. Postoperative SSIs were significantly associated with longer postoperative hospital stays, hypertension, higher American Society of Anesthesia (ASA) scores, longer procedure durations, and the use of a greater number of blood transfusion units.

This study revealed a low SSI rate following spinal surgery. We identified a history of hypertension, prolonged hospitalization, longer operative time, blood transfusion, and higher ASA score as risk factors for SSI in spine surgery in our population. As our findings are from a single institute, we believe that a national research collaboration among multiple disciplines should be performed to provide better estimates of SSI risk factors in our patient population.

## 1 Introduction

### 1.1 Background/rationale

The reported prevalence of surgical site infections (SSIs) in spinal surgery series is highly variable and ranges from 0.5% to 18%.^[1]^ Moreover, according to data from the National Nosocomial Infections Surveillance System, approximately 1.7% and 3.2% of patients who underwent a laminectomy procedure and spinal fusion, respectively, developed an SSI. A study published in 1992 at our institution found that the collective SSI rate was 9%.^[2]^ This complication is a source of significant patient morbidity and is a reason for reoperation, which has substantial implications on healthcare expenditure.

There are a limited number of studies that describe the epidemiology and risk factors for SSIs following spinal surgery in the Saudi population. Therefore, most infection control policies are based on research conducted in the North American setting, which has inherent limitations in external validity.

### 1.2 Objectives

As part of our hospital quality improvement initiative, we conducted this study to estimate the SSI rate in patients who underwent spinal surgery in our local population and identify the associated risk factors. These findings may be unique to our community.

## 2 Materials and methods

### 2.1 Study design and setting

We utilized the medical records from the King Abdul Aziz University Hospital (KAUH) in Jeddah, Saudi Arabia, to conduct a retrospective case–control study. We defined the study outcome as SSIs that occurred after surgery. The study was approved by the Biomedical Ethics Research Committee at the KAUH (reference number: 207-17).

### 2.2 Study participants

Our study included patients treated with adult services (age >14 years), as per our local institutional policies, who underwent spinal surgery at the KAUH between January 2014 and December 2016. All cases treated with pediatric service (age <14 years) were excluded from the study.

The Saudi healthcare system is designed so that patients are consistently returned to the primary hospital where their operation took place so that the treatment of complications at other healthcare facilities is highly unusual. This policy lowered the likelihood of incomplete data on postoperative complications in our study.

### 2.3 Outcome definition and data collection (variables)

We adopted the definition set by the Centers for Disease Control and Prevention for incisional SSIs, which defines an SSI as an event that occurs within 30 days after the procedure (where day 1 is the procedure date), that involves the skin and subcutaneous tissue of the incision, and that occurs in a patient that has at least one of the following:

a) purulent drainage from a superficial incision;
b) an organism(s) identified from an aseptically obtained specimen from the superficial incision or subcutaneous tissue;
c) a superficial incision that is deliberately opened by a surgeon or physician and in which culture or non-culture-based testing of the superficial incision or subcutaneous tissue is not performed, where the patient has at least one of the following signs or symptoms: localized pain or tenderness, localized swelling, erythema, or heat; and
d) a physician's diagnosis of a superficial incisional SSI.^[3]^

Our hospital policies mandate that SSIs are routinely surveilled and, upon confirmation, reported to the infection control department at the KAUH. We started our study by obtaining a list of the unique hospital medical record numbers of consecutive spinal surgery patients who underwent surgery at KAUH between January 2014 and December 2016 from the neurosurgery department. Only 3 neurosurgeons performed such cases in that period. Then, we obtained a list of the patients who developed SSIs during that period from the Infectious Diseases Control Department and linked the 2 lists. We used the electronic and paper-based hospital records from KAUH to extract all of the participants’ data.

We extracted data on demographic characteristics, anthropometric measurements, preoperative laboratory investigations and infection-preventive measures, comorbidities, intraoperative measures, and postoperative care.

### 2.4 Data analysis

To describe our study population, we utilized the frequencies and absolute numbers, and median and interquartile range (IQR) for categorical and continuous variables, respectively. Differences in categorical variables were assessed using the chi-squared or Fisher's exact tests when the data were sparse. The Mann–Whitney *U* test was used to analyze continuous variables (Table 1). When the statistical analysis was performed, a *P*-value < .05 was considered significant. Univariate logistic regression was performed to estimate the odds ratios (ORs) and 95% confidence intervals (CIs) for the association between SSI following spinal surgery and patient characteristics (Table 2). Statistical analysis was performed using SPSS Statistics for Windows version 19.0 (released 2010; International Business Machines Corporation, Armonk, NY).

**Table 1:** Patients characteristics stratified by surgical site infection (SSI).

Variable	Patients without SSI (n = 193)	Patients with SSI (n = 8)	*P*
Age, median by year (IQR)	56.9 (45–69)	68.6 (61.4–70.7)	.14
Gender			.49
Male, n (%)	100 (51.8)	3 (37.5)	
Female, n (%)	93 (48.2)	5 (62.5)	
Nationality			.72
Saudi	123 (63.7)	6 (75)	
Non-Saudi	68 (35.2)	2 (25)	
No. of any missing values	2		
BMI, median (IQR)	27.7 (25.2–31.5)	30.3 (23.5–33.1)	.95
Previous spine surgery			.18
Yes	175 (90.7)	6 (75)	
No	18 (9.3)	2 (25)	
Smoking			.55
Yes	20 (10.4)	1 (12.5)	
No	173 (89.6)	7 (87.5)	
Urgency level			1
Elective	182 (94.3)	8 (100)	
Emergency	11 (5.7)	0 (0)	
Diabetes mellitus			.69
Yes	52 (26.9)	3 (37.5)	
No	135 (69.9)	5 (62.5)	
Unknown	6	–	
Hypertension			.04
Yes	43 (22.3)	5 (62.5)	
No	145 (75.2)	3 (37.5)	
Unknown	5		
ASA class			.06
I	52 (26.9)	1 (12.5)	
II	122 (63.2)	5 (62.5)	
III	17 (8.8)	1 (12.5)	
IV–V	2 (1)	1 (12.5)	
Location of procedure			.16
Cervical	76 (39.4)	0	
Thoracic	10 (5.2)	0	
Lumbar	74 (38.3)	6 (75)	
Sacral	2 (1)	0	
Mixed	31 (16)	2 (25)	
No. of operated vertebrae			.76
1	120 (62.2)	4 (50)	
2–4	60 (31.1)	3 (37.5)	
>4	13 (6.7)	1 (12.5)	
Blood loss			.005
Less than 100 mL	101 (52.3)	0	
More than 100 mL	69 (35.7)	6 (75)	
No. of any missing values	23 (11.9)	2 (25)	
Blood transfusion			<.001
No blood transfusion	172 (89.1)	2 (25)	
1–2 units	15 (7.8)	6 (75)	
More than 3 units	6 (3.1)	0	
Prophylactic antibiotic			.08
Given	65 (33.7)	6 (75)	
Not given	91 (47.1)	2 (25)	
Unknown	37	–	
Preoperative steroid use			1
Yes	22 (11.4)	1 (12.5)	
No	166 (86)	7 (87.5)	
Unknown	5		
Preoperative shaving			.36
Yes	36 (18.6)	8 (100)	
No	157 (81.3)	0	
Subcutaneous drain insertion			.08
Yes	80 (41.4)	6 (75)	
No	113 (58.6)	2 (25)	
Use of implant			.1
Yes	136 (70.5)	0	
No	57 (29.5)	8 (100)	
LOS preoperatively (d), median (IQR)	2 (1–2)	2 (1.5–25.5)	.08
LOS postoperatively (d), median (IQR)	5 (3–7)	17.5 (9–22)	<.001
Procedure duration (min), median (IQR)	170 (102–235)	277.5 (223–303)	.003

ASA = American Society of Anesthesia, IQR = interquartile range.

**Table 2:** Univariate regression analysis assessing factors associated with surgical site infection.

	Univariate	
Variable	OR (95%CI)	*P*
Age	1.03 (0.98–1.09)	.23
Female gender	1.79 (0.42–7.71)	.43
Previous spine surgery	3.24 (0.61–17.25)	.17
Body mass index	0.98 (0.86–1.12)	.78
Smoking	1.44 (0.17–12.59)	.74
Diabetes mellites	1.56 (0.36–6.75)	.55
Hypertension	5.62 (1.29–24.47)	.02
Preoperative hemoglobin level	0.68 (0.48–0.98)	.04
Preoperative white blood cell count	0.99 (0.87–1.14)	.98
Preoperative partial thromboplastin time	1.07 (1.04–1.11)	<.001
Preoperative creatinine level	1.04 (1.02–1.06)	<.001
Preoperative urea level	1.24 (1.02–1.50)	<.03
ASA class		
I	1 Ref	
II–III	2.25 (0.26–19.09)	.50
IV–V	26.0 (1.16–583.46)	.04
Duration of the procedure (min)	1.01 (1.01–1.02)	.01
Length of hospital stay before surgery (d)	1.17 (1.05–1.31)	.004
Postoperative hospital stays (d)	1.05 (1.02–1.09)	.006
Blood transfusion	23.3 (4.43–122.73)	<.001
Prophylactic antibiotics	0.24 (0.05–1.23)	.09
Use of drain	4.16 (0.82–21.16)	.09

ASA = American Society of Anesthesia, CI = confidence interval, OR = odds ratio.

## 3 Results

### 3.1 General study participant characteristics

We included 201 patients who underwent spinal surgery in our study, with a mean follow-up period of 31 months (range, 16–54 months). Their median age was 56.9 years, and 51.2% were men. The overall SSI rate in our study population was 4.0% (n = 8/201). The median body mass index (BMI) was 27.7, and 10.4% of the patients had a history of smoking. Only 27.4% of the patients had diabetes mellitus. None of the patients had bleeding disorders. The majority of patients had an American Society of Anesthesia (ASA) score ≤ 2 (89.6%; n = 180).

Revision surgeries accounted for 10.0% (n = 20) of all procedures performed. The majority of the included cases (94.5%; n = 190) were elective. The cervical and lumbar spine were the most commonly operated levels (37.8% and 39.8%; n = 76 and 80, respectively). Most cases (61.7%; n = 124) underwent single-level surgeries. The majority of the cases had spinal instrumentation inserted (70.5; n = 136).

### 3.2 Study participant characteristics stratified by the SSI group

There were no significant differences in age, sex, nationality, or BMI between the patients who did and did not develop SSIs. The proportion of patients with a history of hypertension was higher in the patients who developed SSIs compared to those who did not (62.5% vs 22.3%, respectively; *P* = .04). The estimated amount of intraoperative blood loss and the number of blood transfusions were significantly different between the 2 groups (*P* = .005 and *P* < .001, respectively). The lengths of postoperative hospital stays were significantly longer among patients who developed SSIs when compared to those who did not (median [IQR], 5 [3–7] days vs 17.5 [9–22] days; *P* < .001). Additionally, the median procedure durations differed significantly (median [IQR], 170 [102–235] minutes vs 277 [223–303] minutes; *P* = .003) (Table 1).

Table 2 shows the factors associated with SSI that were assessed in the univariate regression analysis. History of hypertension was a significant factor associated with SSI. The preoperative laboratory findings of hemoglobin level and elevated partial thromboplastin time, creatinine level, and urea level (OR 5.62, 0.68, 1.07, 1.04, and 1.24; 95% CI 1.29–24.47, 0.48–0.98, 1.04–1.11, 1.02–1.06, and 1.02–1.50, respectively) were statistically significant. Univariate analysis demonstrated that the pre- and postoperative lengths of hospital stay (OR 1.17, 1.05, 95% CI 1.05–1.31 and 1.02–1.09, respectively) were statistically significant factors.

Intraprocedural factors, including the procedure duration and blood transfusion (OR 1.01 and 23.3, 95% CI 1.01–1.02 and 4.43–122.73, respectively), were significantly associated with SSIs.

## 4 Discussion

### 4.1 Key results and interpretation

SSI after spinal surgery is frequently encountered in clinical practice. Its occurrence has been shown to result in increased morbidity and mortality rates and increased healthcare costs.^[4,5]^ A variety of risk factors have been described, including the patient's age, obesity, diabetes, urinary incontinence, tobacco use or a history of smoking, poor nutritional status, complete neurologic deficit, ASA score > 2, previous urinary tract infections (UTIs), hypertension, unintentional durotomies, operating level, revision surgery, nonsteroidal anti-inflammatory drug (NSAID) use, a posterior surgical approach, oncologic cases, increased estimated blood loss, blood transfusion use, increased operative time, extension to the sacrum, and use of spinal instrumentation.^[4–11]^

This study attempted to evaluate the role of the previously mentioned SSI risk factors in our local population despite the standard local infection control measures and policies such as preoperative patient washing, shaving, and prophylactic antibiotic administration. There is a lack of research on infection control and quality improvement in our region. Moreover, the unique demographic characteristics of Saudi Arabia, with the high prevalence of obesity, diabetes, and smoking, might lead to higher SSI rates and different associated risk factors.^[12–14]^

Previous studies have shown that prolonged surgery, prolonged hospital stays, and poor postoperative wound care are likely to result in SSIs.^[15]^ These results are supported by our study, as we found that prolonged operative times increased the chance of developing SSI.

The postoperative infection rate tends to increase among individuals with diabetes and whose hospital stay is prolonged compared to non-diabetics, as shown in previous studies. Our data could not support these findings, as our sample size was small. We propose that a study with a larger sample size may be required to capture such an effect.^[16]^

There is a growing body of evidence associating blood transfusions with SSIs regarding the risks associated with blood transfusion.^[17,18]^ Smoking and obesity are other well-known SSI risk factors, but we could not demonstrate this with our small patient cohort.^[19]^

Our study showed that prolonged operative times could increase the risk of developing an SSI postoperatively. These results are consistent with those found in the study conducted by Hamdeh et al.^[20]^ Further studies showed that the risk of developing an SSI is related to the procedure duration, nutritional status, and patient comorbidities.^[21]^

SSIs can potentially have devastating consequences following spinal surgery; therefore, Cassir et al stated that active surveillance and regular feedback from patients are necessary to reduce the SSI rate.^[22]^

Our study results showed that only 4% of the recruited patients developed SSIs after spinal surgery. In contrast, a study conducted by Hu et al found that 45% of the patients included in the study developed SSIs.^[23]^ The results indicated that the prevalence of SSIs could be decreased by reducing the duration of the patients’ pre- and postoperative hospital stays, in combination with antibiotic prophylaxis administration. These results are consistent with those of this study. The results have shown that an increase in the length of hospital stays significantly increases the risk of developing an SSI.

Another study conducted by Bellusse et al revealed that the risk factors that increased the chances of developing an SSI included a prolonged hospital stay, high BMI, blood transfusions, and the length of surgery.^[24]^ Rao et al conducted a study to elucidate the risk factors for SSI and develop effective strategies to prevent SSI.^[25]^ Their results demonstrated that open suction drains are likely to increase the SSI risk after spinal fusion procedures. Our study findings were consistent with the work conducted by Meng et al. They found that a history of smoking, UTI, diabetes, hypertension, blood transfusion, spinal surgery, and cerebrospinal fluid leakage are risk factors for developing an SSI following spinal surgery.^[6]^

Few studies have found similar results to ours concerning hypertension as a risk factor for SSI. Yao et al and Saeedinia et al found that hypertension was associated with SSI in patients undergoing spinal surgery. However, few hypotheses have been postulated in this regard, including those relating to subcutaneous tissue and skin hypoperfusion in such patients, leading to higher SSI rates.^[26,27]^

One unexpected finding was the low prophylactic antibiotic administration rate in our cohort, as only a minority of our cases received timely prophylaxis. Whether this finding is a factual or a result of insufficient documentation remains unknown. Proper documentation is an essential prerequisite for infection control policy evaluation and improvement. We believe that this finding represents a defect in our hospital system that must be addressed and that we are attempting to improve.

Our study also demonstrates that research collaborations are urgently needed in Saudi Arabia because our conclusions are limited by the findings being restricted by the single-center experience and the small number of spinal surgeries conducted at our hospital. The limited number of patients included in our study likely accounted for our failure to demonstrate a significant association between SSIs and their well-known risk factors.

### 4.2 Study limitations

The retrospective observational design, with its inherent limitations, is a significant weakness of this study. The accuracy of the hospital data was dependent upon the quality of the information entered into the electronic and paper-based medical records. Since spinal surgery is considered a major surgery that requires a high level of documentation and given that most of our study variables were objectively measured, we expected a lower risk of errors related to measurement. Our study's further limitation is that the hospital records did not contain data on potential variables of interest, such as nutritional status, NSAID use, and the type of microorganisms cultured.

Our study is also limited by the small numbers of patients and infections that were included, which could lower the ability to statistically determine further risk factors for SSI and achieve statistical power in a multivariate analysis of the data. Since our study is a single-center study, this may limit its generalizability to Saudi Arabia.

## 5 Conclusions

This study found low SSI rates following spinal surgery. In our population, we identified a history of hypertension, prolonged hospitalization and operative times, blood transfusion, and a high ASA score as risk factors for SSI in spinal surgery. Our findings are derived from a single institute. We believe there is a need for national research collaboration among multiple disciplines to determine better information about SSIs and their risk factors in our patient population.

## Author contributions

**Conceptualization:** Saleh Baeesa.

**Data curation:** Saleh Algamdi, Maha Alawi, Rakan Bokhari, Saleh Baeesa.

**Formal analysis:** Saleh Algamdi, Maha Alawi, Rakan Bokhari, Khalid Bajunaid, Abdelmoniem Mukhtar, Saleh Baeesa.

**Investigation:** Rakan Bokhari, Khalid Bajunaid.

**Methodology:** Saleh Algamdi, Maha Alawi, Rakan Bokhari, Khalid Bajunaid, Abdelmoniem Mukhtar, Saleh Baeesa.

**Project administration:** Saleh Baeesa.

**Resources:** Maha Alawi, Khalid Bajunaid.

**Software:** Abdelmoniem Mukhtar.

**Supervision:** Maha Alawi, Saleh Baeesa.

**Writing – original draft:** Saleh Algamdi, Maha Alawi, Rakan Bokhari, Khalid Bajunaid, Abdelmoniem Mukhtar, Saleh Baeesa.

**Writing – review & editing:** Khalid Bajunaid, Saleh Baeesa.

## Correction

When originally published, Saleh Saad AlGamdi's name appeared incorrectly as Saleh Algamadi. This has been corrected. Saleh Saad AlGamdi's affiliation appeared incorrectly and has now been corrected to “Department of Ophthalmology, faculty of Medicine, imam abdulrahman bin faisal university, dammam, Saudi Arabia.”

## References

[1] Klein JD Garfin SR. Nutritional status in the patient with spinal infection. Orthop Clin North Am 1996;27:33–6. 8539050

[2] Eltahawy A Mokhtar A Khalaf RM. Postoperative wound infection at a University hospital in Jeddah, Saudi Arabia. J Hosp Infect 1992;21:79–83. 1351499 10.1016/0195-6701(92)90156-g

[3] National Healthcare Safety Network C. Surgical Site Infection (SSI) event. http://www.cdc.gov/nhsn/pdfs/pscmanual/9pscssicurrent.pdf. Published 2017 (accessed July 7, 2020).

[4] Wimmer C Gluch H Franzreb M. Predisposing factors for infection in spine surgery: a survey of 850 spinal procedures. J Spinal Disord 1998;11:124–8. 9588468

[5] Fei Q Li J Lin J. Risk factors for surgical site infection after spinal surgery: a meta-analysis. World Neurosurg 2016;95:507–15. 26054871 10.1016/j.wneu.2015.05.059

[6] Meng F Cao J Meng X. Risk factors for surgical site infections following spinal surgery. J Clin Neurosci 2015;22:1862–6. 26282155 10.1016/j.jocn.2015.03.065

[7] Capen DA Calderone RR Green A. Perioperative risk factors for wound infections after lower back fusions. Orthop Clin North Am 1996;27:83–6. 8539055

[8] Dahners LE Mullis BH. Effects of nonsteroidal anti-inflammatory drugs on bone formation and soft-tissue healing. J Am Acad Orthop Surg 2004;12:139–43. 15161166 10.5435/00124635-200405000-00001

[9] Picada R Winter RB Lonstein JE. Postoperative deep wound infection in adults after posterior lumbosacral spine fusion with instrumentation: incidence and management. J Spinal Disord 2000;13:42–5. 10710149 10.1097/00002517-200002000-00009

[10] Glassman SD Dimar JR Puno RM. Salvage of instrumented lumbar fusions complicated by surgical wound infection. Spine 1996;21:2163–9. 8893444 10.1097/00007632-199609150-00021

[11] Blomstedt GC. Infections in neurosurgery: a retrospective study of 1143 patients and 1517 operations. Acta Neurochir (Wien) 1985;78:81–90. 3911746 10.1007/BF01808684

[12] al-nuaim AR al-rubeaan K al-mazrou Y. High prevalence of overweight and obesity in Saudi Arabia. Int J Obes Relat Metab Disord 1996;20:547–52. 8782731

[13] Alqurashi KA Aljabri KS Bokhari SA. Prevalence of diabetes mellitus in a Saudi community. Ann Saudi Med 2011;31:19–23. 21245594 10.4103/0256-4947.75773 PMC3101719

[14] Bassiony MM. Smoking in Saudi Arabia. Saudi Med J 2009;30:876–81. 19617999

[15] Massie JB Heller JG Abitbol JJ. Postoperative posterior spinal wound infections. Clin Orthop Relat Res 1992;284:99–108. 1395319

[16] Simpson JM Silveri CP Balderston RA. The results of operations on the lumbar spine in patients who have diabetes mellitus. J Bone Joint Surg Am Vol 1993;75:1823–9. 10.2106/00004623-199312000-00013 8258554

[17] Ho C Sucato DJ Richards BS. Risk factors for the development of delayed infections following posterior spinal fusion and instrumentation in adolescent idiopathic scoliosis patients. Spine (Phila Pa 1976) 2007;32:2272–7. 17873822 10.1097/BRS.0b013e31814b1c0b

[18] Zhan R Zhu Y Shen Y. Postoperative central nervous system infections after cranial surgery in China: incidence, causative agents, and risk factors in 1,470 patients. Eur J Clin Microbiol Infect Dis 2014;33:861–6. 24306099 10.1007/s10096-013-2026-2

[19] Patel N Bagan B Vadera S. Obesity and spine surgery: relation to perioperative complications. J Neurosurg Spine 2007;6:291–7. 17436915 10.3171/spi.2007.6.4.1

[20] Abu Hamdeh S Lytsy B Ronne-Engstrom E. Surgical site infections in standard neurosurgery procedures – a study of incidence, impact and potential risk factors. Br J Neurosurg 2014;28:270–5. 24588653 10.3109/02688697.2013.835376

[21] Beiner JM Grauer J Kwon BK. Postoperative wound infections of the spine. Neurosurg Focus 2003;15:P1–5. 10.3171/foc.2003.15.3.14 15347232

[22] Cassir N De La Rosa S Melot A. Risk factors for surgical site infections after neurosurgery: a focus on the postoperative period. Am J Infect Control 2015;43:1288–91. 26300100 10.1016/j.ajic.2015.07.005

[23] Hu B-Y Lu X-H Ye J-J. A study on the prevention and management of surgical site infection post spinal surgery. Biomed Res 2017;28:192–8.

[24] Bellusse GC Ribeiro JC Campos FRd. Risk factors for surgical site infection in neurosurgery. Acta Paul Enferm 2015;28:66–73.

[25] Rao SB Vasquez G Harrop J. Risk factors for surgical site infections following spinal fusion procedures: a case–control study. Clin Infect Dis 2011;53:686–92. 21890772 10.1093/cid/cir506

[26] Yao R Zhou H Choma TJ. Surgical site infection in spine surgery: who is at risk? Global Spine J 2018;8: (Suppl): 5S–30S. 10.1177/2192568218799056 PMC6295819 30574441

[27] Saeedinia S Nouri M Azarhomayoun A. The incidence and risk factors for surgical site infection after clean spinal operations: a prospective cohort study and review of the literature. Surg Neurol Int 2015;6:154–154. 26500800 10.4103/2152-7806.166194 PMC4596055

